# Macrophage Migration Inhibitory Factor Induces Autophagy via Reactive Oxygen Species Generation

**DOI:** 10.1371/journal.pone.0037613

**Published:** 2012-05-22

**Authors:** Yung-Chun Chuang, Wen-Hong Su, Huan-Yao Lei, Yee-Shin Lin, Hsiao-Sheng Liu, Chih-Peng Chang, Trai-Ming Yeh

**Affiliations:** 1 Institute of Basic Medical Sciences, Medical College, National Cheng Kung University, Tainan, Taiwan; 2 Research Center of Infectious Disease and Signaling, Medical College, National Cheng Kung University, Tainan, Taiwan; 3 Department of Microbiology and Immunology, Medical College, National Cheng Kung University, Tainan, Taiwan; 4 Department of Medical Laboratory Science and Biotechnology, Medical College, National Cheng Kung University, Tainan, Taiwan; Catholic University Medical School, Italy

## Abstract

Autophagy is an evolutionarily conserved catabolic process that maintains cellular homeostasis under stress conditions such as starvation and pathogen infection. Macrophage migration inhibitory factor (MIF) is a multifunctional cytokine that plays important roles in inflammation and tumorigenesis. Cytokines such as IL-1β and TNF-α that are induced by MIF have been shown to be involved in the induction of autophagy. However, the actual role of MIF in autophagy remains unclear. Here, we have demonstrated that incubation of human hepatoma cell line HuH-7 cells with recombinant MIF (rMIF) induced reactive oxygen species (ROS) production and autophagy formation, including LC3-II expression, LC3 punctae formation, autophagic flux, and mitochondria membrane potential loss. The autophagy induced by rMIF was inhibited in the presence of MIF inhibitor, ISO-1 as well as ROS scavenger N-acetyl-L-cysteine (NAC). In addition, serum starvation-induced MIF release and autophagy of HuH-7 cells were partly blocked in the presence of NAC. Moreover, diminished MIF expression by shRNA transfection or inhibition of MIF by ISO-1 decreased serum starvation-induced autophagy of HuH-7 cells. Taken together, these data suggest that cell autophagy was induced by MIF under stress conditions such as inflammation and starvation through ROS generation.

## Introduction

Autophagy is an active “self-eating” process in which cytoplasmic components are degraded through the endosomal and lysosomal fusion resulting in the formation of autophagosomes [1], [2]. Autophagy enables the cell to survive under various stress conditions, including nutrient starvation, hypoxia, and pathogen infection. In addition, autophagy plays important roles in innate and adaptive immunity, both in the direct elimination of intracellular pathogens and in the processing and presentation of endogenously expressed antigens via major histocompatibility complex antigens [3].

Autophagy begins with the sequestration of an area of the cytoplasm inside a double membrane vesicle called autophagosome [4], [5]. Subsequently, autophagosomes fuse with lysosomes to form autolysosomes, or to late endosomes to give amphisomes [6]. Two ubiquitin-like conjugation of autophagy proteins (ATG5 and ATG12) are essential for autophagosome formation, which promote lipidation of a cytosolic form of light chain 3 (LC3; LC3-I). LC3 is a mammalian homolog of the yeast ATG8 protein that is cleaved and then conjugated to phosphatidylethanolamine to form the LC3-phosphatidylethanolamine conjugate (LC3-II). The lipidated LC3-II is tightly associated with the autophagosomal membranes. Immunoblotting or immunofluorescence staining of LC3 has been commonly used to monitor autophagy where the amount of LC3-II or LC3 punctae formation reflects the existence of autophagosome.

In autophagic process, reactive oxygen species (ROS) is generated through mitochondrial electron transport chains as well as from the cytosol [7], [8]. It is generally believed that accumulation of ROS induces autophagy and causes mitochondria membrane potential loss of the autophagic cells [9], [10]. However, the mechanisms of ROS generation in autophagy are largely unclear. Previous studies have also suggested that cytokines are important regulators of the autophagic process. Thus, T helper type 1 (Th1) cytokines such as IFN-γ, IL-12 and TNF-α induce or promote autophagy in macrophage as well as non-immune cells [11], [12]. In contrast, Th2 cytokines such as IL-4, IL-10 and IL-13 seem to be antagonists of autophagy induction [13].

Macrophage migration inhibitory factor (MIF) is a pluripotent cytokine with enzymatic tautomerase activity, which plays important roles in the modulation of inflammation [14], [15] as well as in cell proliferation, angiogenesis, and tumorigenesis [16]–[20]. MIF is expressed constitutively inside of cells that bind to JAB1 to inhibit activation of JNK and AP1 [21]. Upon various stimuli, cytosolic MIF is released [22]. Once released, MIF binds to cell surface receptor CD74 and the transduce signal augments the secretion of TNF-α and counteracts the anti-inflammatory action of glucocorticoids [23], [24]. Serum levels of MIF are correlated with disease severity in patients with sepsis, cancer, or autoimmune diseases [22], [25]. However, the effect of MIF on cell autophagy is unclear. In this study, we proved that rMIF induces autophagy in human hepatoma cell line HuH-7. In addition, MIF is released during serum starvation of HuH-7 cells. In the presence of MIF inhibitor, ISO-1, or diminished MIF expression by shRNA transfection led to decreased autophagy in these stressed cancer cells.

## Results

### rMIF Induces Autophagy in Human Hepatoma Cells

**Figure 1:**
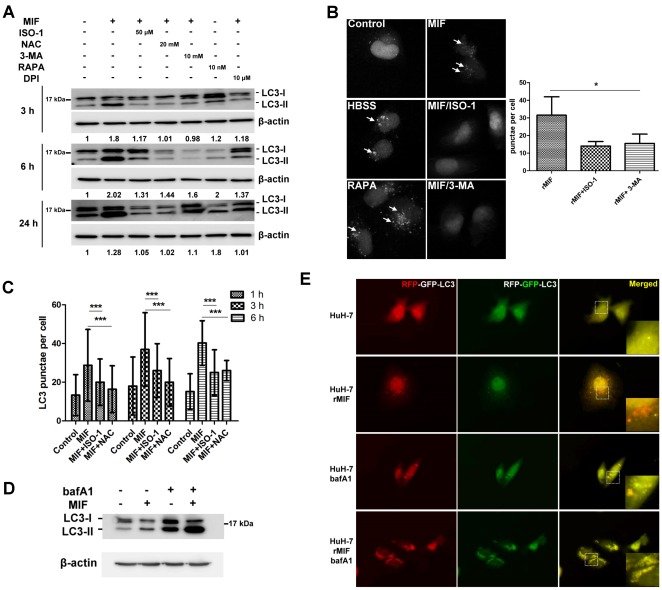
rMIF induces autophagy in human hepatoma cell line HuH-7 cells. (A) HuH-7 cells were treated with rMIF with or without the presence of ISO-1, NAC, 3-MA, DPI, or rapamycin for 3-h, 6-h, and 24-h as indicated. The LC3 conversion was analyzed by Western blotting. The fold of change was listed under each band. The figure represents one of three independent experiments. (B) HuH-7-LC3-EGFP cells were treated with rMIF with or without the presence of ISO-1 (50 µM), 3-MA (10 mM), or rapamycin (10 nM) for 6-h. The cells were fixed, followed by observing punctae formation using ImageExpress Micro system as described in the Materials and Methods. LC3 punctae per cell were quantified 300 cells from three independent experiments using MetaXpress. Negative control was untreated (Control), Positive control was HBSS treated (HBSS). *, P<0.05. Data are means ± SD. (C) HuH-7-LC3-EGFP cells were treated with rMIF with or without the presence of ISO-1 (50 µM), or NAC (20 mM) for 1-h, 3-h, and 6-h as indicated. The LC3 punctae formation were analyzed and quantified as described above. ***, P<0.001. Data are means ± SD. (D) HuH-7 cells were incubated with rMIF and bafilomycin A1 (25 µM) for 24-h. The LC3 conversion was analyzed by Western blotting. The figure represents one of three independent experiments. (E) HuH-7 cells transfected with ptfLC3 plasmid were treated with or without rMIF and bafilomycin A1 (25 µM) for 24-h. The images were monitored by fluorescence microscopy (600× magnification).

We used rMIF to treat a human hepatoma cell line HuH-7 cells to determine if MIF can induce autophagy. Using PI/Annexin V double staining, we found no significant change of cell death in the presence of rMIF for 24-h (data not shown). However, Western blotting analysis of the cell lysates indicated rMIF induced the conversion of the cytosolic LC3-I to LC3-II after 3-h, 6-h, and 24-h of incubation (Fig. 1A). In addition, MIF specific inhibitor ISO-1 reduced LC3-II conversion. Previous studies have shown that 3-MA (an inhibitor of type III PI3K), NAC (a ROS scavenger) inhibits autophagy. Herein, we found 3-MA and NAC showed ability to inhibit MIF-induced LC3-II conversion. In addition, an NADPH oxidase inhibitor, DPI, also inhibited MIF-induced LC3 conversion. Furthermore, pEGFPC1-LC3 plasmid was transfected into HuH-7 cells (HuH-7-LC3-EGFP) to observe EGFP-LC3 punctae formation by fluorescent microscopy. Treatment of rMIF to HuH-7-LC3-EGFP for 6-h resulted in ∼33 punctae formation per cell that was reduced to ∼15 punctae in the presence of ISO-1 or 3-MA (Fig. 1B, 1C). Serum starvation induced by HBSS and rapamycin were used as positive controls here. In addition, in the presence of bafilomycin A1 (a vacuolar ATPase inhibitor that blocks fusion of autophagosome and lysosome), the LC3-II expression was increased in HuH-7 cells treated with or without rMIF (Fig. 1D). These results suggest an increase of autophagic flux in the cells treated with rMIF. To further clarify whether MIF induced autophagic flux, mRFP-GFP tandem fluorescence-tagged LC3 plasmid (ptfLC3) was transfected into HuH-7 cells to monitor the progression of the autophagic flux. The GFP tag is acid sensitive while the RFP tag is acid-insensitive. The double tagged LC3 was used to label autophagosomes, amphisomes, and autolysosomes [6], [26]. In autophagosomes both tags emit fluorescent light resulting in yellow fluorescent dots. However, fusion of autophagosomes to late endosomes or lysosomes results in acidic amphisomes or autolysosomes where the green fluorescence from GFP is lost resulting in red fluorescent dots. After 24-h rMIF treatment, red LC3 punctae were increased in treated HuH-7 cells while more yellow LC3 punctae were found in untreated HuH-7 cells (Fig. 1E). Furthermore, using bafilomycin A1 to block autophagic flux before autophagosome-lysosome fusion, an increase of yellow LC3 punctae was noticed in rMIF treated cells. Therefore, these results indicate that MIF induced an autophagic flux.

### rMIF Stimulates ROS Generation and Causes Mitochondrial Membrane Potential (MMP) Loss

**Figure 2:**
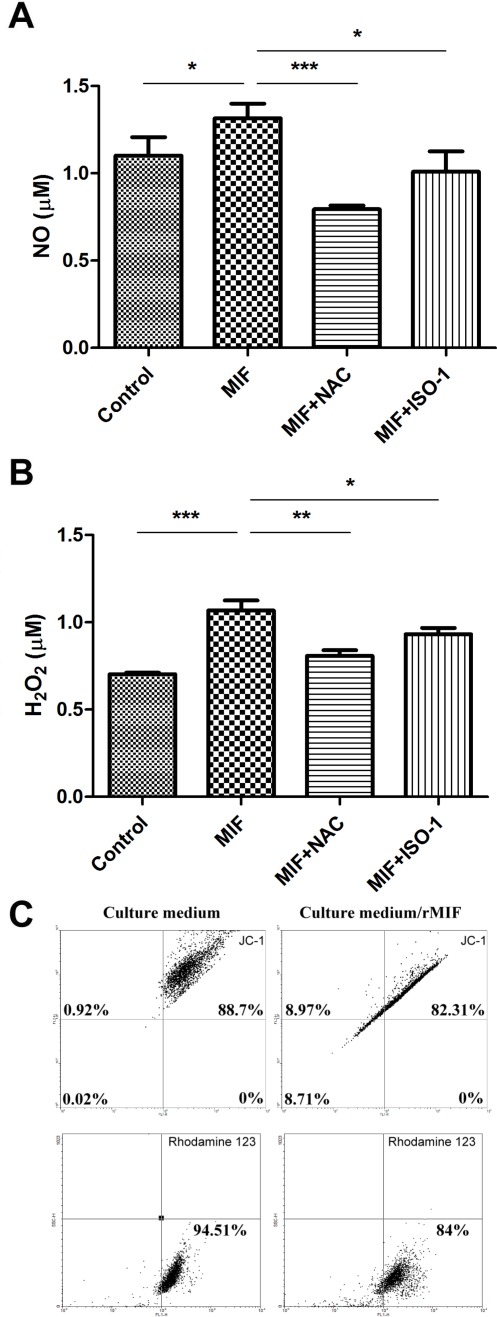
rMIF stimulates ROS generation and causes MMP loss. HuH-7 cells were incubated with rMIF (0.16 nM) with or without the presence of ISO-1 or NAC for 6-h. (A) The NO (B) H_2_O_2_ levels in supernatants were detected as described in the Materials and Methods. (C) The MMP changes in HuH-7 cells were detected using JC-1 and rhodamine 123 after 6-h incubation with rMIF using flowcytometry.

The levels of NO and H_2_O_2_ in supernatants of rMIF-treated HuH-7 cells were shown in Fig. 2. Stimulation of HuH-7 cells with rMIF for 6-h significantly increased NO production from ∼1.1 µM to ∼1.3 µM (p = 0.0255). When NAC or ISO-1 was co-incubated with rMIF, the levels of NO were decreased to ∼0.7 and ∼1 µM, respectively (Fig. 2A). The production of H_2_O_2_ was also increased in, rMIF-treated group that showed an average level around 1.1 µM as compared to ∼0.75 µM in the non-treated group. The increase of H_2_O_2_ production by rMIF treatment was also inhibited in the presence of NAC or ISO-1 (Fig. 2B). In addition, mitochondrial membrane potential (MMP), an indicator of ROS generation, was detected in these cells using JC-1 or rhodamine 123.Higher MMP in normal culture group than rMIF treated group were found (Fig. 2C; Up/Left: JC-1 aggregation; Down/Left: rhodamine 123 uptake). When rMIF were treated, JC-1 exhibited higher green fluorescence than red fluorescence (Fig. 2C; Up/Right). The uptake of rhodamine 123 was also reduced about 10% in the presence of rMIF in the culture medium.

### Serum -starvation Induces MIF Secretion

**Figure 3:**
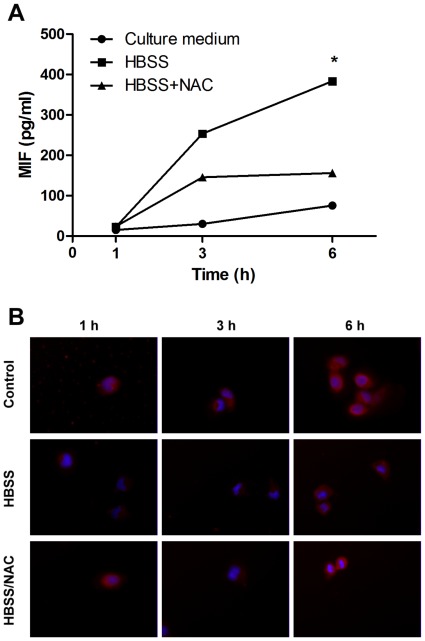
Serum-starvation induces MIF secretion. (A) HuH-7 cells were incubated with HBSS with or without the presence of NAC. The levels of MIF in the supernatants were detected by ELISA after different time of serum starvation as indicated. * P<0.05. Data are means ± SD from three independent experiments. (B) HuH-7 cells were incubated with HBSS with or without NAC for 1-h, 3-h, and 6-h. The cells were immobilized and the distribution of intracellular MIF was detected by immunofluorescence assay.

Since serum starvation can induce cell autophagy through ROS generation, we tested whether MIF is involved in serum-starvation-induced autophagy and ROS generation. We first examined the secretion of MIF during serum-starvation by incubation of cells in HBSS for different periods of time as indicated. As shown in Fig. 3A, serum starvation of HuH-7 cells in HBSS significantly induced MIF release after 3 h, which reached to 400 pg/ml after 6-h. On the contrary, in the control cells, there was only a slight increase of MIF secretion after 6-h of incubation. In the presence of NAC, serum starvation-induced MIF secretion was partly inhibited (Fig. 3A). Immunofluorescent staining of intracellular MIF expression was also decreased 3-h after serum starvation, which was recovered 6-h after serum starvation (Fig. 3B).

### Serum Starvation-induced ROS Generation and Autophagy Formation are Inhibited by MIF Inhibitor, ISO-1

**Figure 4:**
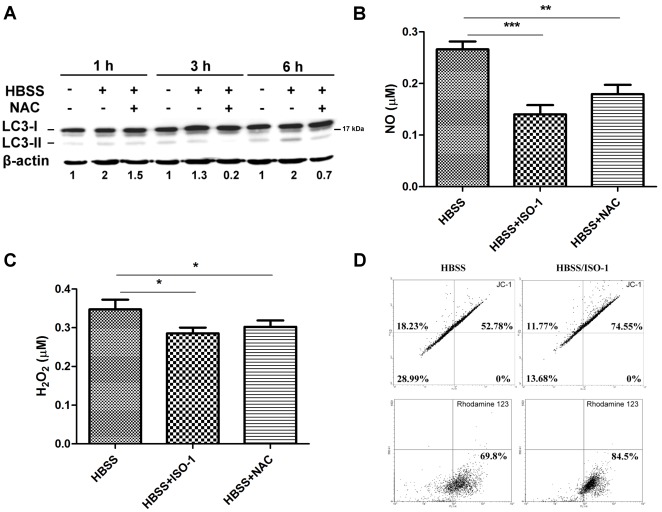
Serum starvation-induced ROS generation and autophagy formation are inhibited by MIF inhibitor, ISO-1. (A) HuH-7 cells were stimulated with starvation by HBSS and incubated with or without ROS scavenger NAC (20 mM) for 1-h, 3-h, or 6-h. LC3 conversion were analyzed by Western blotting. The conversion of LC3-I to lipidated LC3-II was computed and listed under each band. (B) HuH-7 cells were stimulated with starvation by HBSS with or without ISO-1 (20 µM) or NAC for 6-h. The level of NO in supernatants was detected. (C) Supernatants from HBSS-treated HuH-7 cells for 6-h were collected to detect H_2_O_2_ concentration. (D) Mitochondrial membrane potential (MMP) of HuH-7 cells was detected after incubating with HBSS with or without the presence of MIF inhibitor, ISO-1 for 6-h. Upper panel indicated the MMP which was detected by JC-1. Lower panel represents rhodamine 123 uptake by HuH-7 cells. The uptake of JC-1 and rhodamine 123 was detected by flow cytometry.

To further understand whether MIF is involved in serum starvation induced autophagy, we first confirmed HuH-7 cells incubated in HBSS could induce LC3-II conversion at 1-h to 6-h (Fig. 4A). In the presence of ROS scavenger NAC, this starvation-induced autophagy as well as NO and H_2_O_2_ production was inhibited (Fig. 4B, 4C). HBSS-treated group showed ∼0.27 µM of NO and ∼0.35 µM of H_2_O_2_ in supernatants. In the presence of ISO-1, both the levels of NO and H_2_O_2_ production as well as MMP depolarization-induced by serum starvation were decreased significantly (Fig. 4D).

### MIF Depletion Reduces Serum Starvation-induced ROS Generation and MMP Loss

**Figure 5:**
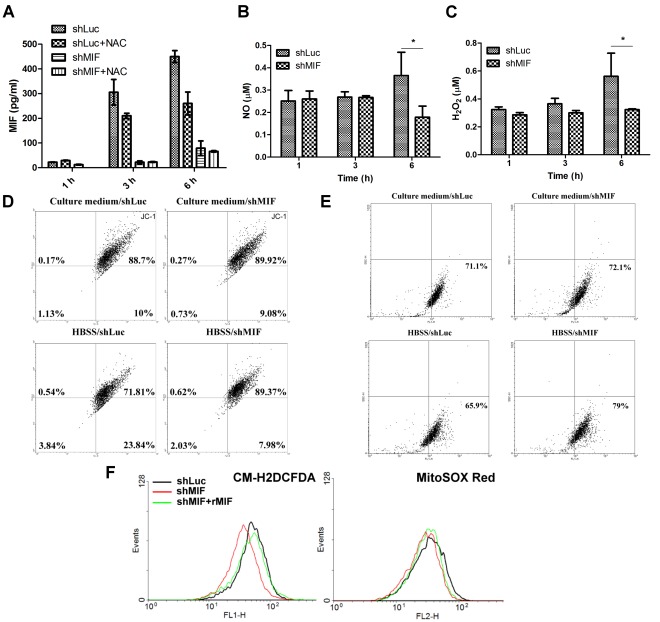
MIF depletion reduces serum starvation-induced ROS generation and MMP loss. (A) The MIF levels in supernatants of HuH-7-shLuc and HuH-7-shMIF cells were detected by ELISA after incubation with HBSS with or without NAC for different periods of time as indicated. (B) The NO levels (C) The H_2_O_2_ levels in supernatants of HuH-7-shLuc and HuH-7-shMIF cells were detected as described in the Materials and Methods after incubation with HBSS for different periods of time as indicated. MMP loss in HuH-7-shLuc and HuH-7-shMIF cells after incubation with HBSS for 6-h were detected by (D) JC-1 or (E) rhodamine 123 as described in the Materials and Methods. (F) HuH-7-shLuc and HuH-7-shMIF cells were incubated with HBSS or with additional rMIF for 1-h followed by detecting superoxide using CM-H2DCFDA and MitoSOX Red dye.

We used lentivirus based shRNA system to knockdown endogenous MIF in HuH-7 cells to further characterize the role of MIF in serum starvation-induced ROS generation. As found in HuH-7 cells, the secretion of MIF in control cells (HuH-7-shLuc) was increased after serum starvation, which was inhibited in the presence of NAC (Fig. 5A). Conversely, HuH-7-shMIF showed low levels of MIF secretion during starvation. Thus, we next detected the NO and H_2_O_2_ production of these two cells during starvation. Both NO and H_2_O_2_ secretion showed no difference between HuH-7-shLuc and HuH-7-shMIF cells during incubation with HBSS at 1-h and 3-h. However, decreases of NO and H_2_O_2_ production in HuH-7-shMIF supernatants was found after 6-h of serum starvation (Fig. 5B, 5C). Serum starvation induced MMP loss of these two cells was also different. In HuH-7-shMIF cells, the red fluorescence intensity was higher than HuH-7-shLuc when JC-1 dye was used (Fig. 5*D*; Down). The uptake of rhodamine 123 showed ∼15% higher in HuH-7-shMIF cells after 6-h starvation (Fig. 5E; Down). To further differentiate the source of superoxide generation in cells, cellular superoxide was detected by CM-H2DCFDA dye, while mitochondrial superoxide was measured by MitoSOX Red. More cellular superoxide generation was found in serum-starvation of HuH-7-shLuc cells than HuH-7-shMIF cells after serum starvation (Fig. 5F). Moreover, when rMIF was added, the cellular superoxide of HuH-7-shMIF cells was also increased. However, only slight increases of mitochondrial superoxide were found when MitoSOX red dye was used.

### Depletion of Endogenous MIF Reduces Starvation-induced Autophagy

**Figure 6:**
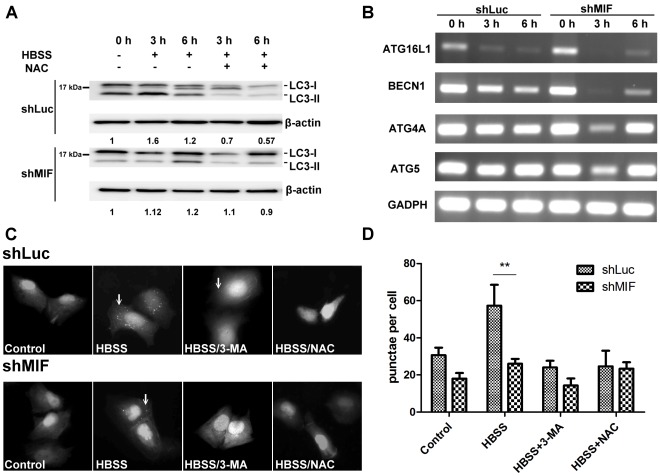
Depletion of endogenous MIF reduces starvation-induced autophagy. (A) HuH-7-shLuc and HuH-7-shMIF cells were incubated with HBSS with or without NAC for 3-h and 6-h. The cell lysates were collected to detect LC3 conversion by Western blotting analysis. The figure represents one of three independent experiments. (B) HuH-7-shLuc and HuH-7-shMIF cells were incubated in HBSS for starvation. The mRNA changes of autophagy-related genes were analyzed at 0-h, 3-h, and 6-h as indicated. (C) LC3 punctae formation in LC3-EGFP transfected HuH-7-shLuc and HuH-7-shMIF cells 6-h after incubation with HBSS was detected as described in the Materials and Methods. (D) The results LC3 punctae formation was calculated by ImageExpress Micro system. **, P<0.01. Data are means ± SD.

LC3-II conversion in HuH-7-shLuc cells but not in HuH-7-shMIF cells confirmed endogenous MIF was required for serum starvation-induced autophagy in HuH-7 cells (Fig. 6A). The mRNA level of autophagy-related gene (ATG) such as ATG16L1, BECN1, ATG4A, and ATG5 were also decreased in HuH-7-shMIF after 3-h and 6-h of starvation (Fig. 6B). In addition, lower punctae formation was found in HuH-7-shMIF-EGFP-LC3 cells compared to control HuH-7-shLuc-EGFP-LC3 cells 6-h after starvation and punctae formations in both cells were all inhibited in the presence of 3-MA or NAC (Fig. 6C and 6D).

## Discussion

Nutrient deprivation such as serum starvation can induce cell autophagy through ROS generation [9], [27]. In addition, several cytokines that are involved in both innate and adaptive immunity induced or inhibited autophagy [28]. In this study, we first demonstrated that rMIF induced autophagy of HuH-7 cells. MIF-induced autophagy of HuH-7 cells were further confirmed using a tandem fluorescent tagged LC3-(mRFP-GFP) reporter plasmid (pffLC3) to study the autophagic flux. Results from bafilomycin A1, a fusion blocker of autophagosome and lysosome suggest an increased LC3II and punctae in rMIF-treated cells due to increased autophagic initiation but not decreased autophagic flux.

Autophagy of HuH-7 cells induced by MIF were inhibited in the presence of ROS scavenger, NAC or NADPH oxidase inhibitor, DPI. These results suggest MIF induced autophagy through ROS generation. Indeed, the generation of ROS by MIF stimulation was confirmed by the detection of NO and H_2_O_2_ in the supernatants of rMIF-treated HuH-7 cells. In addition, MMP depolarization in these cells was also confirmed by JC-1 staining and rhodamine uptake. Therefore, MIF not only induced inflammatory response but also ROS and autophagy of the cells.

Although mitochondria are notorious for ROS production, they are not the only sites of intracellular oxidative stress. ROS can also be generated from the cytosol [8]. To differentiate the source of ROS induced by MIF, we used MitoSOX Red to measure mitochondrial superoxide and CM-H2DCFDA to check cytosolic superoxide. We found mitochondrial superoxide was slightly decreased in HuH-7-shMIF cells after serum starvation as compared with HuH-7-shLuc cells, which was reversed when rMIF was added into HuH-7-shMIF cells. Using CM-H2DCFDA, we found MIF increased cytosolic superoxide generation in HuH-7 cells. Thus, both mitochondrial and cytosolic superoxide in HuH-7 cells are induced by MIF.

Since MIF is constitutive expressed in cells [22], we tested whether endogenous MIF is involved in serum starvation-induced autophagy. We found that intracellular MIF was secreted into supernatant after serum starvation. In addition, serum starvation-induced autophagy was decreased in MIF knockdown cells or in the presence of MIF inhibitor, ISO-1. Thus, these results suggest that the stress induced by serum starvation triggered endogenous MIF release. The released MIF then binds to CD74 in autocrine and paracrine fashions to induce autophagy through ROS generation. It is known from previous reports that MIF-CD 74 complex will be internalized through endocytosis, leading to sustained ERK activation [29]. In addition, endocytosis of activated receptor results in activation of NADPH oxidase and generation of ROS within early endosome [30]. Therefore, CD74-mediated MIF endocytosis contributes to ROS generation and autophagy induction.

Even though the role of MIF in tumorigenesis has been reported in many different cancers, the significance of MIF in term of tumor survival is unclear [16]–[20]. Broadly, stress simultaneously provokes both an adaptive and apoptotic response within cells. Therefore, ROS induces both autophagy and apoptosis [9]. Autophagy is generally considered a pro-survival mechanism protecting cancer cells under stress or poor nutrient conditions while apoptosis will lead to cell death [31], [32]. Consequently autophagy induced by MIF facilitates the development of cancer resistance to chemotherapy-induced cell death. Interestingly, another proinflammatory mediator (high-mobility group box 1, HMGB1), which was also released under stress regulates autophagy and apoptosis in cancer cells [33], [34]. Knockdown of HMGB1 or inhibition of its release leads to predominantly apoptosis and decreased autophagy in stressed cancer cells [32]. Whether MIF-induced autophagy will enhance tumor survival under stress condition and whether MIF inhibition is an alternative therapeutic approach against cancer must be further studied in the future.

In summary, our data suggests that, similar to other damage associated molecular pattern, HMGB1, the release of MIF under stress condition not only plays an important role in inflammatory response but also in autophagy of cells. Taken together, MIF-induced ROS generation and autophagy represents a defense mechanism of cells under different conditions of stress.

## Materials and Methods

### Cells

HuH-7 cells [35], human hepatoma cell line, and HEK 293T cells [36] were cultivated in Dulbecco’s Modified Eagle Medium (DMEM) supplemented with 10% heat-inactivated fetal bovine serum (FBS), 2 mM L-glutamine, and 1% nonessential amino acids in a 37°C humidified incubator with 5% CO_2_. To generate LC3-EGFP or mRFP-GFP tandem fluorescence-tagged LC3 (a gift from Dr. Tamotsu Yoshimori [26]) expressed cells, HuH-7 cells (5×10^5^ cells in 6-cm dish) were transfected with 3 µg pEGFPC1-LC3 or ptfLC3plasmid using PolyJet DNA transfection reagent (Signagen Laboratories, Ijamsville, MD). The cells were selected and maintained in culture medium contained G418 sulfate (400 µg/ml). MIF or luciferase (Luc) control knockdown HuH-7 cells were performed as previously described [37]. In brief, lentiviruses were generated from short-hairpin RNA plasmid (MIF: TRCN0000056818; Luc: TRCN0000072243; National RNAi Core Facility, Academia Sinica, Taipei, Taiwan), pMD.G, pCMVDR8.91 co-transfected HEK 293T cells. HuH-7, HuH-7-LC3-EGFP cells were infected with lentivirus and selected in culture medium contained puromycin (2 µg/ml).

### Reagents

4′, 6-diamidino-2-phenylindole (DAPI), Hanks’ Balanced Salt solution (HBSS), rhodamine 123, N-acetyl-L-cysteine (NAC), diphenyliodonium chloride (DPI), JC-1, rapamycin, and 3-methylamphetamine (3-MA) were purchased from Sigma-Aldrich (St. Louis, MO). MIF inhibitor (ISO-1) was obtained from Calbiochem (La Jolla, CA). Human MIF ELISA kit was purchased from R&D Systems (Minneapolis, MN). Recombinant human MIF (rMIF) was prepared as previously described [37].

### Western Blotting

To detect LC3 conversion, 5×10^5^ of HuH-7 cells were cultivated on 6-well plates (GeneDirex, Las Vegas, NV) and incubated with HBSS, rapamycin (10 nM), or rMIF (0.16 nM in culture medium) with or without the presence of different inhibitors such as 3-MA (10 mM), ISO-1 (20 µM), DPI (10 µM), or NAC (20 mM) for 1-h, 3-h,6-h, or 24-h as indicated. Whole cell lysates were harvested using RIPA buffer (GE Health Care, Piscataway, NJ) and separated by 12% SDS-PAGE. The SDS-PAGE were further transferred onto a PVDF membrane and blocked by 5% skim milk in phosphate-buffered saline (PBS). Antibodies against LC3 (MBL International, Woburn, MA) or β-actin (Sigma-Aldrich) were incubated with membrane at 4°C overnight and washed with PBST (0.05% Tween 20 in PBS). The membranes were further incubated with horseradish peroxidase (HRP) conjugated goat anti-rabbit or rabbit anti-mouse IgG (Invitrogen, Camarillo, CA) and detected using Enhanced Chemiluminescence Western Blotting kit (Amersham Pharmacia Biotech, UK). The conversion of LC3-II to LC3-I were measured by Image J software and normalized with the level of β-actin.

### MIF ELISA

HuH-7 cells (5×10^5^ cells/well) were seeded on 6-well plates. The cells were incubated with 1 ml HBSS with or without NAC (20 mM) for 1-h, 3-h, and 6-h as indicated. The supernatants were collected and the levels of MIF were detected by human MIF ELISA (R&D Systems). The absorbance value at 450 nm was read by a VersaMax microplate reader (Molecular Devices, Sunnyvale, CA).

### Reverse-transcription PCR (RT-PCR)

HuH-7 cells were incubated with HBSS for 3 h and total RNA was extracted using total RNA mini kit (Bioman Scientific, Taiwan). Reverse-transcription of total RNA was performed using MMLV (Invitrogen). Specific primers for RT-PCR of autophagy-related genes were as follows: BECN1 (5′-CCATGCAGGTGAGCTTCGT-3′ and 5′- GAATCTGCGAGAGACACCATC -3′), ATG16L1 (5′ AACGCTGTGCAGTTCAGTCC-3′ and 5′- AGCTGCTAAGAGGTAAGATCCA-3′), ATG5 (5′- AAAGATGTGCTTCGAGATGTGT-3′ and 5′- CACTTTGTCAGTTACCAACGTCA-3′), ATG4A (5′- GGAACGGTTAATGACCAGACTT-3′ and 5′- CTGGAGGTACAAAGGGAGGC-3′), GADPH (5′- AAGGTGAAGGTCGGAGTCAAC-3′ and 5′- GGGGTCATTGATGGCAACAATA-3′). PCR were performed using Taq DNA polymerase (Violet BioScience, Taiwan) and analyzed with 2% agarose gel.

### LC3-EGFP, ptfLC3 Punctae and Immunofluorescent Assay (IFA)

For LC3-EGFP and ptfLC3 punctae formation analysis, HuH-7 cells (10^4^ cells) were seeded onto 96-well optical bottom plates (Costar Corning, NY) or eight well chamber slides (Nunc Lab-Tek II-CC2, Nalge Nunc International, Naperville, IL). The cells were incubated with HBSS, rapamycin, or rMIF in the presence of different inhibitors as described above for different time points. The cells were washed by PBS and immobilized with 2% paraformaldehyde containing DAPI (1 µg/ml) for 10-min on ice. The punctae formation of the cells was monitored by ImageExpress Micro system (Molecular Devices, CA) with 40X objective lens or a fluorescence microscope (Leica Geosystems AG, St. Gallen, Switzerland) with 60X objective lens. The images were further analyzed and semi-automatically quantified punctae using MetaExpress software. For IFA, HuH-7 cells were incubated with HBSS with or without NAC for 1-h, 3-h, and 6-h. The cells were immobilized by paraformaldehyde and permeabilized with 0.1% TritonX-100 for 5-min on ice. The cells were incubated with rabbit anti-MIF antibodies (Santa Cruz Biotechnology, Inc., Santa Cruz, CA) at 4°C overnight and detected by Alexa-594 conjugated goat anti-rabbit IgG antibody (Invitrogen).

### Nitric Oxide (NO), Hydrogen Peroxide (H_2_O_2_), and Superoxide Detection

ROS generation was monitored by detecting NO and H_2_O_2_ formation. In brief, HuH-7 cells (5×10^5^ cells/well) were cultivated on 6-well plates and incubated with HBSS and rMIF (0.16 nM) with or without the presence of ISO-1 (20 µM) or NAC (20 mM) in a final volume of 1 ml for 1-h, 3-h, or 6-h. The supernatants were collected to measure NO and H_2_O_2_ level using NO colorimetric assay kit (BioVision; Mountain View, CA) and H_2_O_2_ assay kit (BioVision). For superoxide detection, intracellular and mitochondrial superoxide was determined using CM-H2DCFDA dye (5 µM; Invitrogen) and MitoSOX Red (5 µM; Invitrogen), respectively. HuH-7-shLuc and HuH-7-shMIF cells were incubated with HBSS for 1-h followed by incubated with CM-H2DCFDA or MitoSOX Red for 30-min. The uptake of dye were measured by flowcytometry.

### Mitochondrial Membrane Potential (MMP) Assay

To detect MMP changes, cells (5×10^5^ cells/well) were detected by two different dyes. JC-1 (2 µM) and rhodamine 123 (5 µM) were diluted in DMEM base and incubated for 30-min at 37°C. For JC-1 and rhodamine 123 detection, the cells were analyzed by a FACScan flow cytometer (BD Biosciences, Mountain View, CA). The data were further analyzed by WinMDI 3.0 software.

### Statistical Analysis

Data were expressed as mean ± SD of three independent experiments. Student’s *t* test was analyzed for the significance of the difference between the test and the control groups. A p-value <0.05 was considered significant.
